# Right inferior phrenic artery to right pulmonary artery fistula causing hemothorax: A case report

**DOI:** 10.1016/j.radcr.2024.04.086

**Published:** 2024-05-18

**Authors:** Anthony D'Angelo, Hasan Khan, Garrett Coleman, Irfan Masood

**Affiliations:** a John Sealy School of Medicine, The University of Texas Medical Branch, 301 University Blvd, Galveston, TX 77555, USA; b Department of Radiology, The University of Texas Medical Branch, 301 University Blvd, Galveston, TX 77555, USA

**Keywords:** Inferior phrenic artery to pulmonary artery fistula, Case report, Hemothorax, Embolization

## Abstract

Inferior phrenic artery to pulmonary artery fistulae are a rare anomaly seen on CT thorax angiogram when evaluating for certain pulmonary pathological conditions. A 79-year-old man with hemothorax on chest X-ray was evaluated by interventional radiology for embolization of a bleeding vessel. During the procedure, a fistulous connection between the right inferior phrenic artery and right pulmonary artery with signs of extravasation was found and embolized, significantly reducing the size of the hemothorax. This case demonstrates that inferior phrenic artery to pulmonary artery fistulae, a rarely seen vascular anomaly, can result in life-threatening hemothorax.

## Introduction

Systemic artery to pulmonary artery fistulae (SA-PAF) are a rare anomaly usually seen on CT angiogram of the chest when looking for causes of conditions such as hemoptysis, cough, or ischemic stroke [1,2]. SA-PAF were first described in 1947 [3] and were initially treated with surgical resection and vascular ligation. More recently, SA-PAF can be repaired by embolizing the fistula intravascularly [1,2]. We report a case of inferior phrenic artery to right pulmonary artery fistula that resulted in a large hemothorax with subsequent embolization by interventional radiology.

## Case presentation

**Fig. 1:**
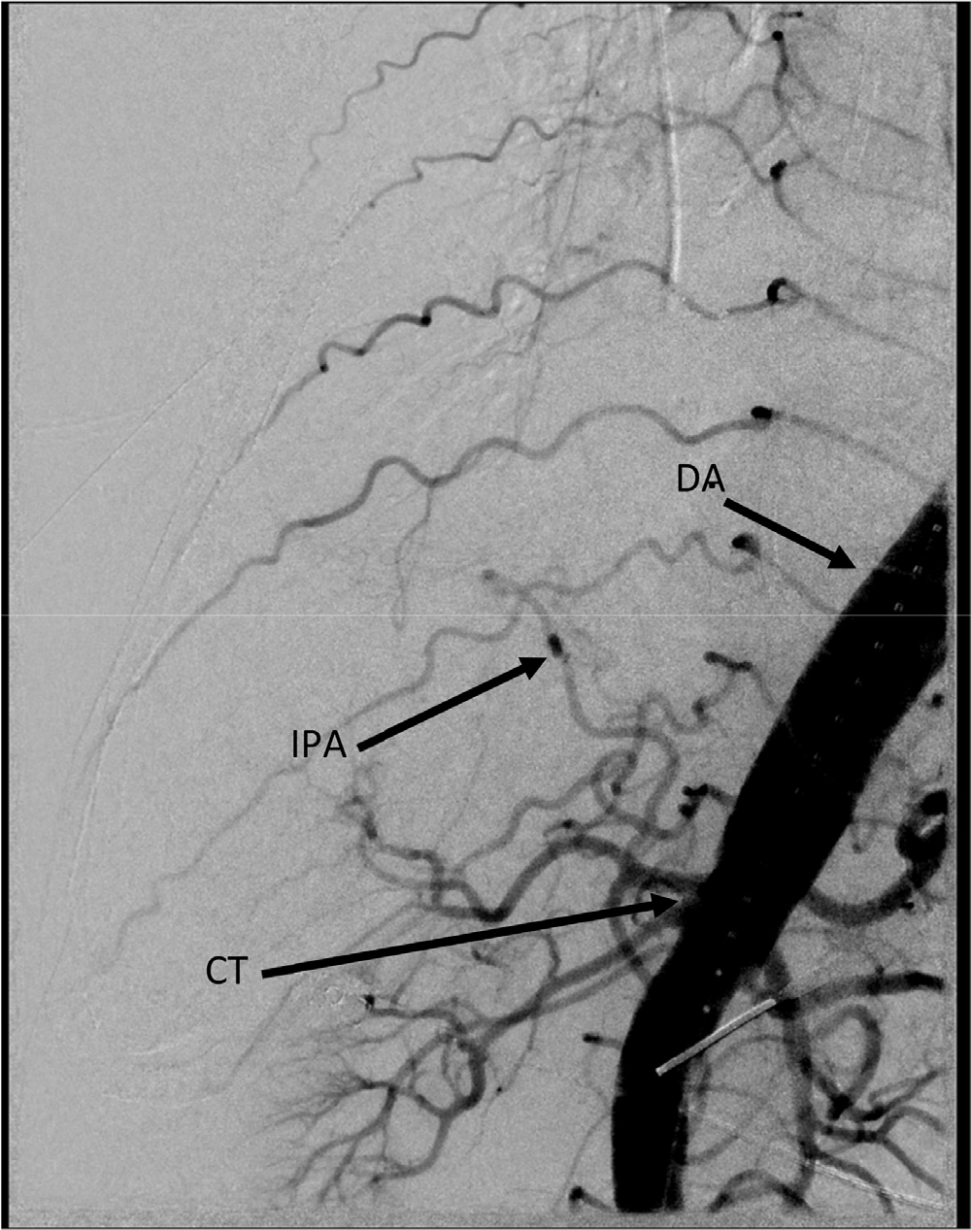
Arteriogram showing right inferior phrenic artery coming off celiac trunk. DA, descening aorta; IPA, inferior phrenic artery; CT, celiac trunk.

**Fig. 2:**
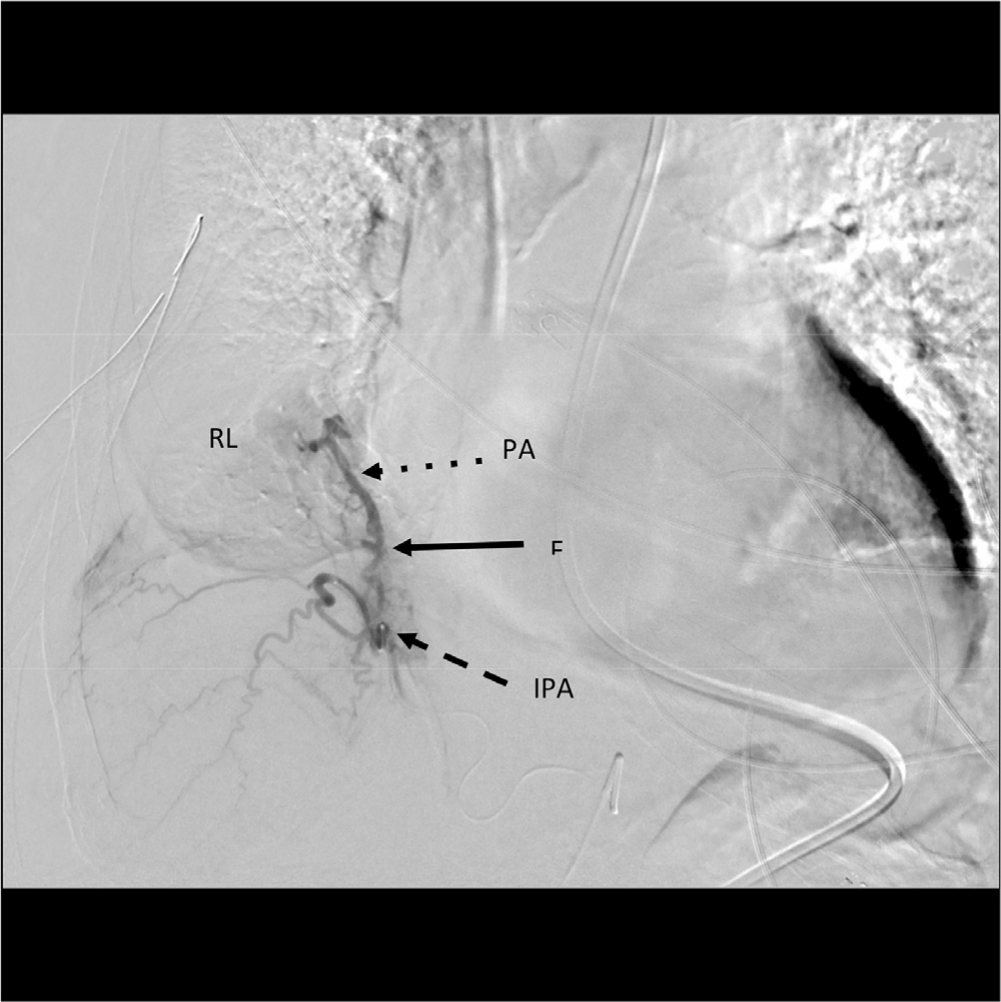
Contrast through right inferior phrenic artery showing fistula through diaphragm to right pulmonary artery branch. RL, right lung; PA, pulmonary artery; F, fistula; IPA, inferior phrenic artery.

A 79-year-old patient with PMH of recent COVID-19 infection initially presented to the emergency department for syncope. Chest X-ray revealed closed fractures of ribs 8-11, and the patient was hospitalized for recovery. On day 2 of hospitalization, elevated D-dimer was seen on blood work. CT thorax with contrast showed a right lower lobe third-order pulmonary embolism and the patient was placed on enoxaparin. On day 6 of hospitalization, the patient's O_2_ saturation dropped to ∼80%, and repeat chest X-ray revealed a large pleural effusion of the right lung. Two chest tubes were placed, which drained 3.7 liters of sanguinous fluid over 24 hours. At this point, the patient was transferred to our tertiary center for advanced care and evaluation, with interventional radiology consulted for possible embolization. The patient was catheterized via ultrasound-guided access to the right common femoral artery. The thoracic aorta, right 11th intercostal, subcostal, first lumbar, and celiac arteries were initially catheterized with subsequent diagnostic runs. Both inferior phrenic arteries originated from the celiac artery (Fig. 1), and further examination of the right inferior phrenic artery revealed an abnormal fistulous communication to a branch of the right pulmonary artery (Fig. 2).

**Fig. 3:**
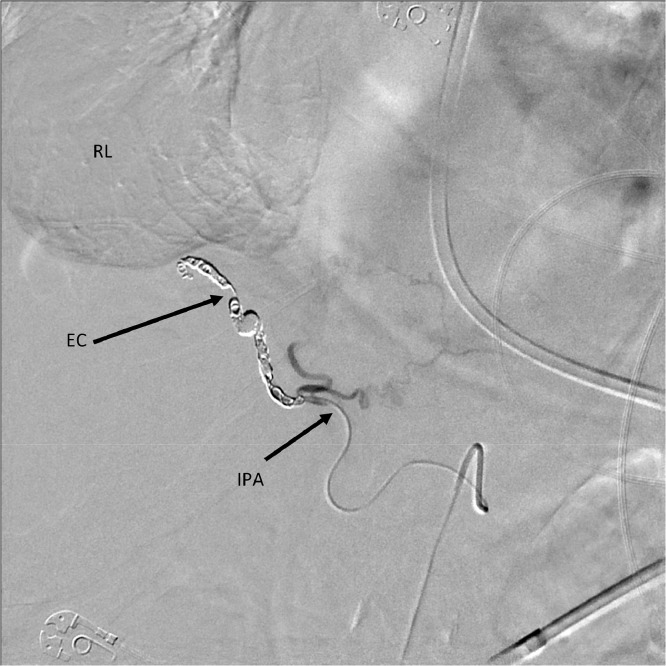
Embolization of right phrenic artery to right pulmonary artery fistula using coiling. RL, right lung; EC, embolization coil; IPA, inferior phrenic artery.

This fistula was embolized (Fig. 3) and the patient's chest tubes showed significant reduction in serosanguinous drainage post-procedurally. One of the tubes was removed 3 days following the procedure. On day 13 of hospitalization, vascular surgery placed an IVC filter to reduce the risk of PE from DVT. Unfortunately, the patient became hemodynamically unstable shortly following IVC filter placement and passed away after signing a DNR.

## Discussion

The first reported case of an SA-PAF was by Burchell et al, who described tortuous vessels connecting to the pulmonary circulation in 1947. At the time, surgical resection of the involved lung lobe was done, with ligation of the connecting systemic arteries to prevent hemorrhage [3]. More recent cases described by Wynne et al, Shimmyo et al, Juergens et al, and Kawakado et al all describe various SA-PAF presentations and their repair using endovascular embolization [1], [2], [3], [4], [5]. Wynne et al describes a right inferior phrenic artery to right pulmonary artery fistula found incidentally while evaluating for cause of ischemic thalamic stroke, with embolization done to reduce the risk of cor pulmonale [2]. Shimmyo et al describe finding a chest wall systemic artery to pulmonary artery and pulmonary vein connection in a 19-year old patient with a history of pneumothorax treated surgically; based on the patient history, they concluded the SA-PAF was secondary to previous pneumothorax surgeries [4]. Juergens et all describe a right inferior phrenic artery to right pulmonary artery fistula in a woman with chronic dyspnea; 2 separate embolizations were performed due to the systemic side having connections with the right lateral thoracic, hepatic, and inferior epigastric arteries [5]. Kawakado et al. describe finding a left inferior prhenic artery to left pulmonary artery fistula in a man with acute cough, with embolization done to prevent future development of hemoptysis [1].

A unique aspect of our case is the presence of hemothorax associated with SA-PAF. A literature search for fistula-related hemothorax usually described arteriovenous fistulae in the pulmonary circulation and previous papers discussing SA-PAFs do not appear to mention hemothorax as a complication [[6], [7], [8]]. Given its presence and the significant reduction in chest tube fluid volume after embolization, it is reasonable to deduce that the SA-PAF was the source of bleeding into the pleural space.

It is not certain what caused the patient's fistula to begin leaking into the pleural space. One possibility is traumatic damage when the patient initially fell which resulted in a slow bleed. This seems unlikely though given that the patient did not start presenting with signs of hemothorax until 6 days post-admission. A more likely possibility is that the bleeding began on the fifth or sixth day of the hospital course. Perhaps the patient's fall resulted in initial physical strain to the fistulous connection that, throughout hospitalization, became progressively weaker until finally rupturing into the pleural space. As seen in this case though, embolization is an effective method of preventing further bleeding into the pulmonary space.

## Conclusion

Inferior phrenic artery to pulmonary artery fistula can result in life-threatening hemothorax should it extravasate into the pleural space. Fistula embolization can be an effective method of reducing hemothorax size.

## Patient consent

Written informed consent was obtained from the patient for the publication of this case report.

## Authorship

The authors declare that this is their original work and they all approve the content of this manuscript. They confirm that this manuscript has not been published previously, in any language, in whole or in part, and is not currently under consideration elsewhere.

## Ethical clearance

This project did not involve any research and no ethical clearance was required.
